# Extracellular matrix-degradable polymer nanostimulants elicit potent immune responses in orthotopic pancreatic cancer via sono-activatable dual-drug synergism

**DOI:** 10.1016/j.mtbio.2025.101954

**Published:** 2025-06-06

**Authors:** Meng Li, Danling Cheng, Yue Wang, Chongwen Xuan, Viktar Abashkin, Jindong Xia, Ling Ding, Jingchao Li

**Affiliations:** a State Key Laboratory of Advanced Fiber Materials, College of Biological Science and Medical Engineering, Donghua University, Shanghai, 201620, China; b Department of Radiology, Songjiang Hospital Affiliated to Shanghai Jiao Tong University School of Medicine, Shanghai, 201600, China; c Institute of Biophysics and Cell Engineering of NASB, 27 Akademicheskaya St., 220072, Minsk, Belarus

**Keywords:** Polymer nanoparticles, Sonodynamic therapy, Immunotherapy, Orthotopic pancreatic cancer, Tumor microenvironment

## Abstract

Pancreatic cancer is a highly aggressive malignancy with a poor prognosis due to its complex tumor microenvironment (TME), which includes a dense extracellular matrix (ECM) and immunosuppressive pathways. Nanomedicine capable of achieving profound tumor penetration and modulating the tumor immune microenvironment is urgently needed to enhance the efficacy of cancer therapy. Herein, we introduce ECM-degradable semiconducting polymer nanostimulants (SPNs) as a novel nanostimulant for deep tumor penetration and multifaceted remodeling of the tumor microenvironment. The SPNs were constructed by loading two immune drugs: toll-like receptor 7/8 agonist (R848) and indoleamine 2,3-dioxygenase inhibitor (NLG919), onto singlet oxygen (^1^O_2_)-responsive SPNs, and modifying their surface with hyaluronidase (HAase). Upon accumulation at orthotopic pancreatic tumor sites, HAase-mediated degradation of the ECM significantly enhances the penetration of nanomedicine into the tumor and facilitates the infiltration of immune cells. Upon sono-activation, the SPNs produce ^1^O_2_, which is not only used for sonodynamic therapy of deep-seated pancreatic tumors and, but also induces immunogenic cell death (ICD) in tumor cells. Simultaneously, the generated ^1^O_2_ can be cleaved by ^1^O_2_-responsive fragments, disrupting the nanoparticle structure and enabling controlled and precise release of the two immune drugs at the tumor site, thus minimizing off-target effects. Through such a multifaceted remodeling mechanism, SPN-based treatment triggers a potent antitumor immunological response. Consequently, the growth of orthotopic pancreatic tumors in mouse models is nearly inhibited, and tumor metastases are effectively suppressed. This study presents an ECM-degradable semiconducting polymer nanostimulant for multifaceted remodeling of the tumor microenvironment, enabling effective and precise immunotherapy of deep-seated orthotopic tumors.

## Highlights

• Extracellular matrix-degradable nanostimulants enable deep tumor penetration and tumor microenvironment remodeling.
• Precise drug release at the tumor site can be achieved by nanostimulants through sono-activation.
• Sono-activatable dual-drug synergy elicits potent immune responses in orthotopic pancreatic cancer.
• This strategy enables effective and precise immunotherapy of deep-seated orthotopic tumors.

## Graphical abstract

**Figure:**
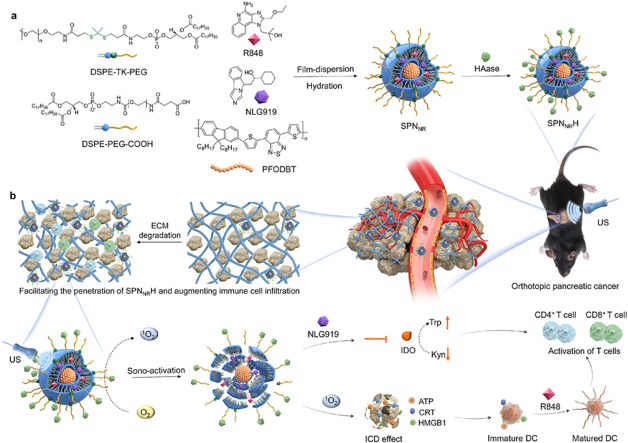

## 1 Introduction

Pancreatic cancer stands as a paradigm of therapeutic resistance among malignancies, with a 5-year survival rate of less than 13 %, which drops to a mere 3 % when distant metastasis occurs [1]. A hallmark of the pancreatic cancer microenvironment is its densely packed extracellular matrix (ECM), characterized by elevated concentrations of hyaluronic acid (HA) and collagen fibers [2]. This ECM not only drives tumor initiation, progression, invasion, and immune evasion but also acts as a physical barricade, severely impeding the penetration of therapeutic agents into the tumor parenchyma [[2], [3], [4]]. Despite multimodal therapies (surgery, chemotherapy, and radiotherapy), outcomes remain dismal due to late diagnosis and inherent treatment resistance [[5], [6], [7], [8], [9]]. A key obstacle is the ECM dual role as a physical barrier and an immunosuppressive modulator within the tumor microenvironment (TME) [10,11]. HA, a dominant ECM component, is strongly associated with tumor invasiveness and metastasis [[11], [12], [13]]. Notably, enzymatic HA degradation using hyaluronidase (HAase) improves chemotherapeutic efficacy by enhancing drug penetration and alleviating interstitial pressure [[14], [15], [16]]. Thus, targeting the ECM while reprogramming the immunosuppressive TME represents a promising strategy to overcome therapeutic resistance and improve survival.

Sonodynamic therapy (SDT) has emerged as a highly promising modality in recent years, leveraging sono-activatable nanoparticles to generate reactive oxygen species (ROS) under ultrasound (US) irradiation [[17], [18], [19], [20]]. Particularly, SDT enables deep tissue penetration and precisely focuses energy on targeted tumor sites, thereby triggering immunogenic cell death (ICD) in tumor cells [[21], [22], [23], [24], [25]]. This cascade of events not only stimulates robust antitumor immune responses but also minimizes collateral damage to adjacent normal tissues, enhancing the overall therapeutic efficacy and safety profile [26,27]. However, the therapeutic outcomes of SDT are often compromised by the complex immunosuppressive TME and apoptosis-resistant mechanisms inherent to cancer cells [23,24]. These factors can significantly undermine the effectiveness of SDT, highlighting the need for complementary strategies to enhance its therapeutic potential.

Immunotherapy has fundamentally reshaped cancer treatment by harnessing and enhancing the body's own immune cells to elicit a powerful systemic anti-tumor response [[28], [29], [30]]. This innovative approach not only effectively eradicates primary tumors but also targets distant metastases, offering comprehensive therapeutic benefits [[31], [32], [33]]. Moreover, immunotherapy holds the promise of establishing long-term immune memory, which can help to suppress the recurrence of cancer [5,34,35]. Despite these advancements, the benefits of immunotherapy for pancreatic cancer remain limited due to its highly immunosuppressive TME [10,35,36]. In addition, systemic biodistribution of these drugs poses safety issues, which might lead to the use of less-than-ideal doses or, in some cases, prevent their use in treating pancreatic cancer altogether [37]. Advancements in nanoparticle-based delivery systems have demonstrated potential to improve cancer immunotherapy through the effective transport of immune drugs to tumor sites, T cells, and circulating dendritic cells (DCs) [28,34,[38], [39], [40], [41], [42]]. Given these, the integration of SDT with nanoparticle-based immunotherapy emerges as a compelling strategy, which not only enhances the delivery of immune drugs but also leverages the deep tissue penetration and precise targeting capabilities of SDT, thereby overcoming the limitations posed by the immunosuppressive TME.

**Fig. 1:**
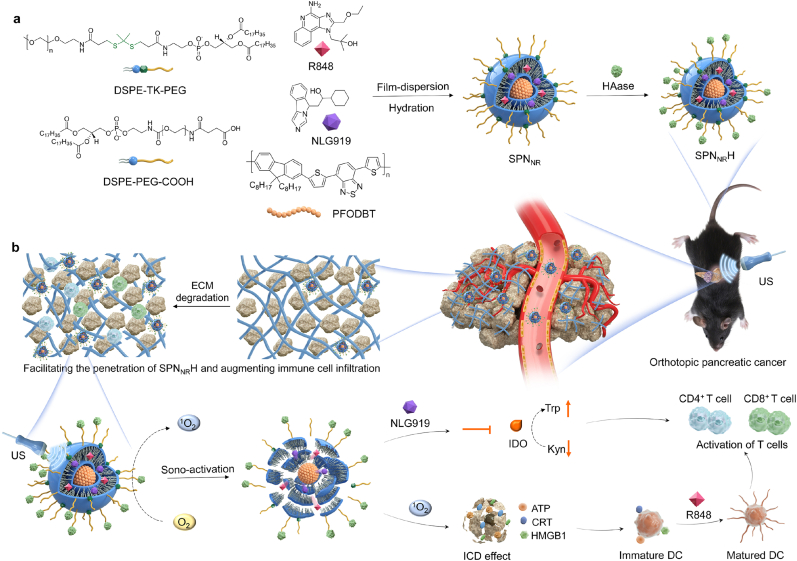
Schematic of sono-activated immunotherapy for pancreatic cancer with ECM-degrading nanostimulants. (a) Design and fabrication of SPN_NR_H nanostimulants employing film-dispersion, hydration, and modification. (b) A schematic illustration clarifies the multifaceted mechanistic pathways of SPN_NR_H in orthotopic pancreatic cancer therapy. This includes degradation of the tumor ECM, enhanced SDT, potentiated ICD, stimulation of antitumor immunity, and regulation of immune suppression, collectively achieving comprehensive remodeling of the tumor microenvironment.

In this study, we designed singlet oxygen (^1^O_2_)-responsive semiconducting polymer-based nanostimulants (SPNs) to achieve multifaceted remodeling of the TME (Fig. 1). We incorporated the immunomodulatory agents NLG919, an indoleamine 2,3-dioxygenase 1 (IDO-1) inhibitor [43] and R848, a TLR7/8 agonist [44], into the SPNs, which are encapsulated with the semiconducting polymer poly[2,7-(9,9-di-octyl-fluorene)-alt-4,7bis(thiophen-2-yl)benzo-2,1,3-thiadiazole] (PFODBT) functioning as a sonosensitizer. PFODBT exhibits stable fluorescence properties and can generate ^1^O_2_ upon stimulation by US radiation, which is crucial for the therapeutic mechanism of SPNs [10]. To further improve therapeutic efficacy, the surface of the SPNs is functionalized with HAase (Fig. 1a). As shown in Fig. 1b, degradation of HA in the tumor microenvironment by HAase boosts the accumulation of nanostimulants and facilitates the infiltration of T cells into orthotopic pancreatic tumor sites. R848 is a potent TLR7/8 agonist that activates DCs and T cells, thereby promoting an immune response and enhancing antitumor immunity [[45], [46], [47]]. NLG919 inhibits the expression of IDO-1, which converts tryptophan (Trp) into kynurenine (Kyn), thus modulating the immunosuppressive TME in pancreatic cancer by increasing the proportion of DCs and T cells while reducing the proportion of regulatory T cells (Tregs) [43,48]. Upon sono-activation, the SPN_NR_H nanostimulants produce ^1^O_2_, which not only facilitates SDT of deep-seated pancreatic tumors and induces ICD in tumor cells but also triggers the controlled release of R848 and NLG919, thereby activating DCs and inhibiting IDO. This comprehensive strategy effectively remodels the TME and elicits a robust antitumor immune response, resulting in near-complete inhibition of orthotopic pancreatic tumor growth and metastasis.

## 2 Materials and methods

### 2.1 Materials

The PFODBT was sourced from Sigma-Aldrich (USA). All ELISA kits were purchased from Solarbio (Beijing, China). All solvents were supplied by Sinopharm (China). The TLR7/8 agonist R848 and IDO inhibitor NLG919 was procured from MedChemExpress (USA). The culture media were purchased from Procell (China)

### 2.2 Synthesis of SPN_NR_H, SPN_NR_ and SPN_N_H

The ^1^O_2_-responsive polymer 1,2-distearoyl-sn-glycero-3-phosphoethanolamine- thioketal-(polyethylene glycol) (DSPE-TK-PEG) was synthesized following a previously reported method [49]. PFODBT, DSPE-TK-PEG, DSPE-PEG-COOH, R848, and NLG919 were dissolved together in chloroform at a mass ratio of 0.25:10:20:0.25:0.25. The mixture was then sonicated to achieve uniform dispersion. Subsequently, the chloroform was removed via rotary evaporation to form a thin film containing PFODBT and the drugs. This film was hydrated in ultrapure water at 55 °C with stirring for 1 h. The hydrated solution was then extruded through 200 and 100 nm membranes using a liposome extruder. After purification by ultrafiltration and washing with water, SPN_NR_ was obtained. To obtain the final product SPN_NR_H, SPN_NR_ was reacted with HAase overnight at 4 °C. The preparation method for SPN_N_H is similar to that of SPN_NR_H, except that R848 is omitted in the initial step.

### 2.3 Characterization of nanostimulants

The particle size and surface charge of SPN_NR_, SPN_N_H, and SPN_NR_H were measured by a Malvern Zetasizer (Nano-ZS90). A UV–Vis spectrophotometer was used to measure their UV–Vis absorption spectra, and a fluorescence spectrophotometer was employed to evaluate the fluorescence properties of the samples.

### 2.4 ^1^O_2_ generation assessment

The production of ^1^O_2_ was evaluated using singlet oxygen sensor green (SOSG). The production of ^1^O_2_ by SPN_NR_, SPN_N_H, and SPN_NR_H (concentration = 20 μg/mL) was measured after US irradiation for 2, 4, 6, 8, and 10 min.

### 2.5 Evaluation of R848 and NLG919 release

To evaluate the US-activated release of R848 and NLG919 by SPN_NR_H, PBS solutions of SPN_NR_H were treated with US for 0, 5, 10, and 15 min. The release of R848 and NLG919 before and after laser irradiation was investigated using the LC-16 SHIMADZU high-performance liquid chromatography (HPLC) method.

### 2.6 In vitro cytotoxicity analysis

Panc02 cells were incubated with varying concentrations of SPN_NR_, SPN_N_H, and SPN_NR_H for 24 h, after which cell viability was evaluated using the CCK-8 assay. In a subsequent experiment, Panc02 cells were incubated with SPN_NR_, SPN_N_H, and SPN_NR_H at a concentration of 35 μg/mL for 12 h, followed by US treatment (1 W/cm^2^, 5 min). Cell viability was then assessed using CCK-8 kit.

### 2.7 In vitro cellular uptake analysis

Panc02 cells were treated with SPN_NR_, SPN_N_H, and SPN_NR_H for 12 h. Subsequently, the cells were trypsinized, washed three times with PBS, and then resuspended in 500 μL of PBS for flow cytometer analysis.

### 2.8 Evaluation of intracellular ROS generation

Panc02 cells were treated with SPN_NR_, SPN_N_H, and SPN_NR_H for 12 h, followed by incubation with H_2_DCFH-DA for 20 min. Afterward, the cells were subjected to US irradiation. Finally, the cells were washed three times with PBS and visualized using a fluorescence microscope.

### 2.9 In vitro spheroid penetration assessment

To evaluate the penetration of nanostimulants, multicellular Panc02 spheroids were constructed and exposed to SPN_NR_, SPN_N_H, and SPN_NR_H (50 μg/mL). Confocal fluorescence microscopy was employed to capture images of these spheroids at various depths (30, 60, 90, 120, 150 and 180 μm).

### 2.10 In vitro Trp metabolism

The methods reported in previous studies were employed to evaluate the Trp metabolism and spheroid penetration of Panc02 cells [49]. Panc02 cells were first treated with interferon-γ (IFN-γ) for 24 h. Subsequently, PBS, SPN_NR_, SPN_N_H, and SPN_NR_H were introduced into the cell culture medium at a final concentration of 20 μg/mL, followed by an additional 24-h incubation period. For the US irradiation group, cells were treated with US (1 W/cm^2^, 5 min) and then cultured for another 24 h. After these treatments, the supernatant from each well was collected, and the concentrations of Trp and Kyn were determined using HPLC.

### 2.11 In vitro ICD characterization and assessment

Panc02 cells were treated with SPN_NR_, SPN_N_H, or SPN_NR_H for 12 h and then subjected to US irradiation treatment. The cells were subsequently placed in an incubator for overnight culture. The following day, the levels of Adenosine Triphosphate (ATP) and high-mobility group box 1 (HMGB1) in the cell supernatant were detected using ELISA kits, respectively. Calreticulin (CRT) expression was assessed by immunofluorescence staining, and the corresponding fluorescence images were obtained with a confocal microscope.

### 2.12 Intratumoral HA level detection

To detect the HA content in orthotopic pancreatic Panc02 tumors, the tumors were removed from mice and then injected intravenously with PBS, SPN_NR_, SPN_N_H, or SPN_NR_H (200 μg/mL, 0.2 mL) through the tail vein. Subsequently, the tumor tissues were placed in a PBS solution with pH = 7.4, ground into a cell suspension, and the HA content was measured using a mouse HA ELISA kit.

### 2.13 In vivo fluorescence imaging and biodistribution study

A solution of SPN_NR_, SPN_N_H, or SPN_NR_H (200 μg/mL, 0.2 mL) was injected intravenously into orthotopic pancreatic cancer mouse models via the tail vein. Using the IVIS fluorescence imaging system, the mice were imaged at 0, 6, 12, 24, and 36 h post-injection (excitation wavelength 520 nm, emission wavelength 700 nm) to observe the dynamic accumulation of nanostimulants in the orthotopic pancreatic tumor. At 36 h post-injection, the C57BL/6 mice bearing tumors were euthanized, and the corresponding tissues were removed and further analyzed for biodistribution using the IVIS fluorescence imaging system.

### 2.14 Detection of intratumoral ROS generation in vivo

To detect the generation of ROS in pancreatic tumors, PBS, SPN_NR_, SPN_N_H, or SPN_NR_H were intravenously injected into orthotopic pancreatic cancer mouse models via the tail vein. After 24 h, the mice were intraperitoneally injected with H_2_DCFH-DA solution (5 μM, 100 μL), and the tumor sites were irradiated with US for 10 min. Subsequently, the ROS levels within the tumors were assessed using fluorescence imaging techniques.

### 2.15 In vivo assessment of antitumor and antimetastatic efficacy

Orthotopic pancreatic cancer mice were intravenously injected with PBS, SPN_NR_, SPN_N_H, or SPN_NR_H (concentration 200 μg/mL, 200 μL) via the tail vein. At 24 h post-injection, the orthotopic pancreatic tumors were treated with US for 10 min. On days 0, 7, and 14, the mice were intraperitoneally injected with D-luciferin potassium salt solution (concentration 20 mg/mL, 150 μL). To monitor tumor growth in real time, bioluminescence (BL) images were acquired using the IVIS imaging system, and the images were analyzed using Living Image software. Subsequently, the tissues from the mice were harvested for ex vivo bioluminescence imaging to evaluate the antimetastatic effect. Meanwhile, the orthotopic pancreatic tumor tissues were collected, weighed, and photographed for documentation.

### 2.16 Monitoring of mouse survival rate

After different treatments, the survival of orthotopic pancreatic cancer mice (n = 5 per group) was monitored daily for 25 days.

### 2.17 In vivo evaluation of immune response

After subjecting the mice to various treatment regimens, they were humanely euthanized. Subsequently, the tumor-draining lymph nodes and orthotopic pancreatic tumors were harvested to generate single-cell suspensions. These suspensions were then stained with fluorescently labeled antibodies. Flow cytometry was employed to analyze the stained cells, enabling the assessment of the immune response.

### 2.18 Statistical analysis

Data were presented as mean ± SD, with sample sizes (n) specified. Significant differences were denoted by ∗(p < 0.05), ∗∗(p < 0.01), and ∗∗∗(p < 0.001). Statistical significance was assessed using a two-tailed unpaired *t*-test, and analyses were conducted with GraphPad Prism 8.0.

## 3 Results and discussion

### 3.1 Sonodynamic and drug release properties of nanostimulants

**Fig. 2:**
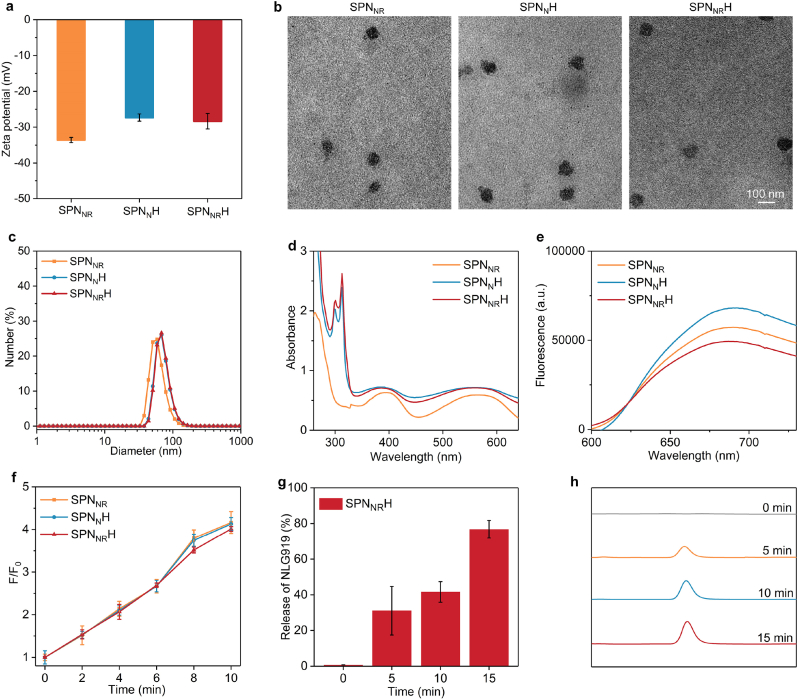
Characterization of the sonodynamic and drug release properties of nanostimulants. (a) Zeta potential measurements of SPN_NR_, SPN_N_H, and SPN_NR_H in aqueous solutions. b) Morphology characterization of SPN_NR_, SPN_N_H, and SPN_NR_H using TEM. (c) DLS size of SPN_NR_, SPN_N_H, and SPN_NR_H. (d) Absorbance spectra of SPN_NR_, SPN_N_H, and SPN_NR_H. (e) Fluorescence emission spectra of SPN_NR_, SPN_N_H, and SPN_NR_H. (f) The ^1^O_2_ generation efficacies of SPN_NR_, SPN_N_H, and SPN_NR_H (n = 3). (g) Release profiles of R848 inhibitors from SPN_NR_H after US irradiation at different time points (n = 3). (h) Release percentages of NLG919 from SPN_NR_H after US irradiation at various time points (n = 3). Data are expressed as mean ± SD.

The SPN_NR_ nanostimulants were synthesized by encapsulating semiconducting polymer (PFODBT), R848 and NLG919 within a ^1^O_2_-responsive shell using thin film dispersion method. Subsequently, HAase was conjugated onto the surface of SPN_NR_ to form the nanostimulants (SPN_NR_H). The zeta potential measurements revealed values of −33.6 mV for SPN_NR_, −27.3 mV for SPN_N_H, and −28.3 mV for SPN_NR_H (Fig. 2a). The protein content in SPN_N_H and SPN_NR_H was significantly higher compared to that in SPN_NR_ (Fig. S1), confirming the successful surface modification with HAase. The nanostimulants (SPN_NR_, SPN_N_H and SPN_NR_H) exhibited a spherical shape and a consistent size distribution (Fig. 2b). The hydrodynamic diameters were 61.31 nm for SPN_NR_, 71.43 nm for SPN_N_H and 72.35 nm for SPN_NR_H (Fig. 2c), indicating that HAase successfully modified the surface of the nanostimulants and that all nanostimulators exhibited good colloidal behavior potential. The optical properties of the nanostimulants were then characterized using UV–vis absorption and fluorescence emission spectra. Similar absorbance peaks at 398 nm and 554 nm, characteristic of PFODBT, were observed for SPN_NR_, SPN_N_H and SPN_NR_H (Fig. 2d). Additional absorbance peaks at around 320 nm were detected for SPN_N_H and SPN_NR_H, attributed to the surface-modified HAase. As shown in Fig. 2e, all nanostimulants exhibited a fluorescence emission peak at 690 nm. These results confirmed that the nanostimulants retained comparable absorbance and fluorescence characteristics, which confirmed successful encapsulation and surface modification.

Further exploration was conducted into the sonodynamic effect and the drug release characteristics of SPN_NR_H. SOSG was employed as an indicator of ^1^O_2_. Solutions containing SPN_NR_, SPN_N_H, and SPN_NR_H exhibited a time-dependent increase in fluorescence intensities (Fig. S2), verifying the production of ^1^O_2_ through the sonodynamic effect of the semiconducting polymer under US irradiation. Following 2, 4, 6, 8, and 10 min of US irradiation, the fluorescence intensities increased by roughly 1.5-, 2.1-, 2.7-, 3.5-, and 4.0-fold, respectively (Fig. 2f). The ^1^O_2_-responsive shells, containing thioketal bond (TK) linked PEG, were designed to facilitate drug release on demand upon US irradiation. In the absence of US irradiation, the release percentage of NLG919 from SPN_NR_H was minimal but increased to 31.1 % after 5 min, 41.6 % after 10 min, and 76.7 % after 15 min of US irradiation (Fig. 2g). Likewise, without US irradiation, negligible release of R848 from SPN_NR_H was observed. In contrast, significant release of R848 was observed after 5, 10, and 15 min of US irradiation (Fig. 2h). These findings indicated that US irradiation activates the release of R848 and NLG919 from SPN_NR_H. Specifically, when subjected to US irradiation, SPN_NR_H produces ^1^O_2_, which degrades the ^1^O_2_-responsive shells, leading to the controlled release of the drugs. Importantly, minimal hemolysis of erythrocytes was observed after incubation with SPN_NR_, SPN_N_H, and SPN_NR_H (Fig. S3), confirming their biocompatibility and suitability for systemic administration.

### 3.2 In vitro anticancer activity and penetrating capability evaluation

**Fig. 3:**
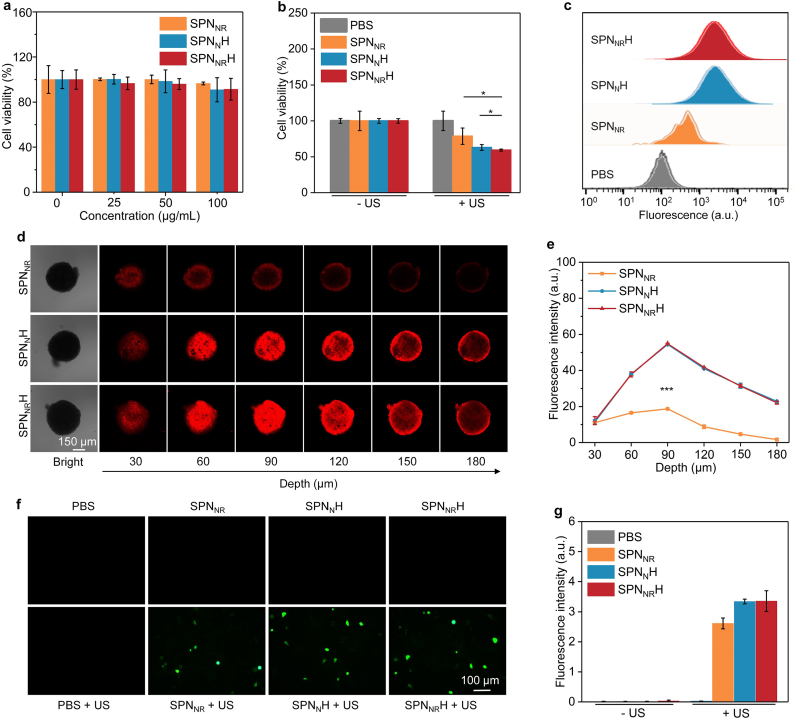
Evaluation of anticancer efficacy and penetrating capability. (a) Cell viability of Panc02 cells treated with SPN_NR_, SPN_N_H, and SPN_NR_H (n = 3). (b) Cell viability of Panc02 cells treated with SPN_NR_, SPN_N_H, and SPN_NR_H under US irradiation (n = 3). (c) Cellular internalization of SPN_NR_, SPN_N_H, and SPN_NR_H via a flow cytometer in Panc02 cells analyzed by flow cytometry. (d) Fluorescence images of 3D multicellular tumor spheroids treated with fluorescence-labeled SPN_NR_, SPN_N_H, and SPN_NR_H at different depths. (e) Fluorescence intensity in 3D multicellular tumor spheroids after treatment at various depths (n = 3). (f) Fluorescence images of Panc02 cells with generation of ROS. (g) Fluorescence intensity of ROS signals in treated Panc02 cells (n = 5). Data are expressed as mean ± SD. Statistical significance was determined using a two-tailed unpaired *t*-test, ∗p < 0.05 and ∗∗∗ (*p* < 0.001).

The cytotoxicity of SPN nanostimulants was first assessed to determine their intrinsic effects on cancer cells. Panc02 cancer cells treated with SPN_NR_, SPN_N_H, and SPN_NR_H at various concentrations exhibited cell viabilities similar to those of untreated control cells (Fig. 3a), indicating minimal cytotoxicity of the nanostimulants themselves. To evaluate in vitro anticancer efficacy, cells treated with the SPN nanostimulants were subjected to US irradiation. Following US exposure, the viability of cells treated with SPN_NR_, SPN_N_H, and SPN_NR_H decreased, with a more pronounced reduction observed at higher SPNs concentrations (Fig. 3b). At a concentration of 35 μg/mL, cell viability was significantly reduced to 78.7 %, 62.9 %, and 59.3 % for SPN_NR_, SPN_N_H, and SPN_NR_H group, respectively. Flow cytometry was employed to investigate the cellular internalization of the nanostimulants. Panc02 cells treated with SPN_N_H, and SPN_NR_H exhibited stronger fluorescence signals compared to those treated with SPN_NR_ and the PBS group (Fig. 3c). These findings indicated that the HAase modification enhances the cellular internalization of the SPN nanostimulants.

To investigate the degradation of the ECM and the subsequent enhancement of nanostimulants penetration, three-dimensional (3D) multicellular tumor spheroids were constructed. At equivalent depths, the red fluorescence signals of nanostimulants in the SPN_N_H, and SPN_NR_H-treated groups were significantly stronger than those in the SPN_NR_-treated group (Fig. 3d). Specifically, at depths of 90, 120, 150, and 180 μm, the fluorescence intensity in the SPN_N_H, and SPN_NR_H groups was approximately 2.5-, 2.7-, 3.9-, and 10.3-fold higher, respectively, compared to the SPN_NR_ group (Fig. 3e). These findings indicated that the surface modification of SPNs with HAase significantly improves nanostimulants penetration within the cell spheroids.

The production of ^1^O_2_ via SDT was verified by fluorescence imaging using DCFH-DA as a probe. Green fluorescence, indicative of ROS signals, was exclusively detected in groups treated with SPN_NR_ + US, SPN_N_H + US, and SPN_NR_H + US -treated groups (Fig. 3f), thereby confirming the production of ^1^O_2_. Notably, the ROS fluorescence intensity was higher in the SPN_N_H + US, and SPN_NR_H + US groups than in the SPN_NR_ + US group. Relative to the PBS control group, the ROS signal intensity increased by 104.0-, 133.6-, and 134.2-fold for the SPN_NR_ + US, SPN_N_H + US, and SPN_NR_H + US groups, respectively (Fig. 3g). These findings confirmed ^1^O_2_ generation via the SDT effect.

### 3.3 In vitro ICD effect evaluation

**Fig. 4:**
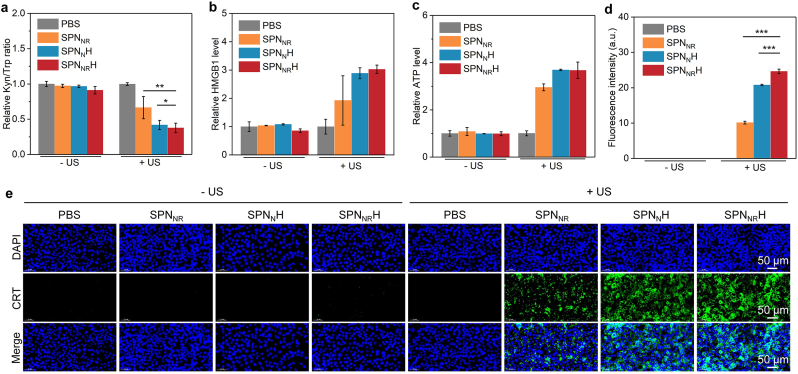
In vitro ICD effect evaluation. (a) Kyn/Trp ratio in panc02 cells treated with SPN_NR_, SPN_N_H, and SPN_NR_H. (*n* = 5). (b) HMGB1 secretion levels in panc02 cells treated with SPN_NR_, SPN_N_H, and SPN_NR_H. (*n* = 5). (c) ATP secretion levels in panc02 cells treated with SPN_NR_, SPN_N_H, and SPN_NR_H. (*n* = 5). (d) Fluorescence intensity of CRT staining signals in panc02 cells treated with SPN_NR_, SPN_N_H, and SPN_NR_H. (*n* = 5). (e) Confocal fluorescence images of CRT staining in panc02 cells treated with SPN_NR_, SPN_N_H, and SPN_NR_H. Data are expressed as mean ± SD. Statistical significance was determined using a two-tailed unpaired *t*-test, ∗ (*p* < 0.05), ∗∗ (*p* < 0.01), and ∗∗∗ (*p* < 0.001).

The in vitro ICD effect induced by nanostimulants treatment and US irradiation was investigated. The ability of SPN_NR_H to inhibit IDO activity was assessed by measuring the extracellular levels of Kyn and Trp. The Kyn/Trp ratio was significantly reduced in the SPN_NR_ + US, SPN_N_H + US, and SPN_NR_H + US groups, whereas no significant changes were observed in the groups treated with nanostimulants alone (SPN_NR_, SPN_N_H, and SPN_NR_H) (Fig. 4a). These results indicated that sono-activation of SPN_NR_, SPN_N_H, and SPN_NR_H effectively inhibits IDO activity, thereby modulating the levels of Kyn and Trp.

The secretion levels of HMGB1 were significantly increased in the SPN_NR_ + US, SPN_N_H + US, and SPN_NR_H + US groups (Fig. 4b). Similarly, the secretion levels of ATP were increased by 5.1-, 5.6-, and 6.7-fold, respectively, in these groups (Fig. 4c). No significant changes in HMGB1 and ATP secretion levels were observed in cells treated with nanostimulants alone, without US irradiation. Confocal microscopy images showed strong green fluorescence signals indicative of CRT exposure on the cell surface in the SPN_NR_ + US, SPN_N_H + US, and SPN_NR_H + US groups, which were markedly distinct from those in the groups treated with nanostimulants alone (Fig. 4e). Quantitative analysis revealed a 4.3-, 7.7-, and 9.4-fold increase in CRT fluorescence intensity in the SPN_NR_ + US, SPN_N_H + US, and SPN_NR_H + US groups, respectively (Fig. 4d). These results demonstrate that SPN_N_H and SPN_NR_H-mediated SDT effectively induces ICD, as evidenced by increased secretion levels of HMGB1 and ATP, and enhanced CRT exposure. These findings confirmed the excellent therapeutic efficacy of SDT in inducing ICD and highlight its potential for in vivo applications.

### 3.4 In vivo TME modulation evaluation

**Fig. 5:**
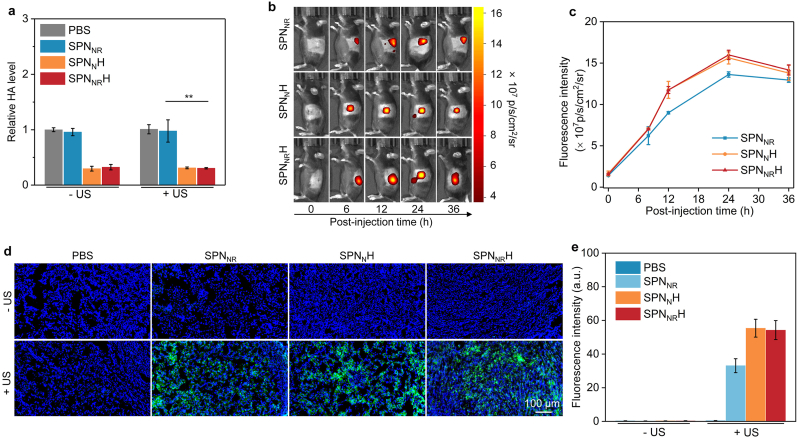
In vivo tumor microenvironment modulation evaluation. (a) Content of HA levels in tumors across different treatment groups. (b) In vivo fluorescence images of Panc02 orthotopic pancreatic cancer bearing mice following intravenous (i.v.) injection of SPN_NR_, SPN_N_H, and SPN_NR_H. (c) Quantitative analysis of fluorescence intensity in orthotopic pancreatic tumors for each group at various time points (*n* = 3). (d) Confocal fluorescence images of the generated ^1^O_2_ in orthotopic pancreatic tumors for each treatment group. (e) Fluorescence intensity of generated ROS signals in orthotopic pancreatic tumors (*n* = 5). Data are expressed as mean ± SD. Statistical significance was determined using a two-tailed unpaired *t*-test, ∗∗ (*p* < 0.01).

The ability of the SPN_NR_H in degrading the tumor ECM and modulating the TME was initially confirmed by evaluating the levels of HA and the accumulation of nanostimulants at the tumor sites. Specifically, the HA levels in orthotopic pancreatic tumor tissues were significantly lower in the SPN_N_H, SPN_NR_H, SPN_N_H + US, and SPN_NR_H + US groups compared to the PBS, PBS + US, SPN_NR_, SPN_NR_ + US groups (Fig. 5a), confirming that the surface-conjugated HAase effectively degraded HA within the tumor site. Additionally, the fluorescence intensity in tumors was higher for SPN_N_H and SPN_NR_H compared to SPN_NR_ (Fig. 5b), with the fluorescence intensity in the SPN_N_H, and SPN_NR_H groups was approximately 1.2-fold higher than that in the SPN_NR_ group (Fig. 5c). These results indicated that SPN_NR_, SPN_N_H, and SPN_NR_H were effectively delivered to orthotopic pancreatic tumor tissues, likely through in situ tumor-generated extracellular vesicle-mediated transport and the enhanced permeability and retention (EPR) effect [[50], [51], [52], [53]]. Furthermore, SPN_N_H and SPN_NR_H demonstrated improved tumor accumulation efficacy due to the surface-conjugated HAase, which degraded the dense ECM, thereby promoting nanostimulants diffusion and penetration.

Subsequently, SDT-mediated ROS generation in the context of HA degradation was investigated. Consistent with the above findings, ROS were detected exclusively in the SPN_NR_ + US, SPN_N_H + US, and SPN_NR_H + US treated groups, with SPN_N_H + US and SPN_NR_H + US exhibiting superior ROS production compared to SPN_NR_ + US (Fig. 5d and e). These results collectively demonstrate that the SPN_NR_H nanostimulants effectively degrade the tumor ECM through HAase-mediated HA degradation, enhancing tumor accumulation and ROS generation upon US activation, thus highlighting their potential for modulating the TME and improving antitumor outcomes.

### 3.5 Deep-tissue orthotopic pancreatic cancer therapeutic and anti-metastasis efficacy evaluation

**Fig. 6:**
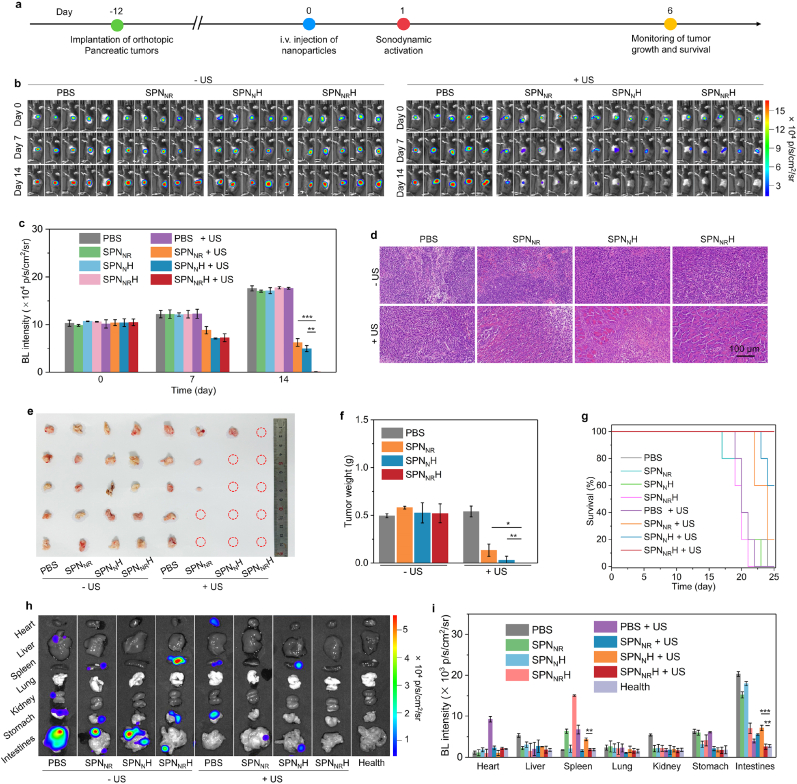
Deep-tissue orthotopic pancreatic cancer therapeutic and anti-metastasis efficacy evaluation. (a) Illustration of the therapeutic efficacy evaluation for deep-tissue orthotopic pancreatic cancer via i.v. injection of SPN_NR_, SPN_N_H, and SPN_NR_H and US followed by US irradiation. (b) In vivo BL imaging analysis of Panc02-Luc orthotopic pancreatic cancer-bearing mice on day 0, 7, and 14 (*n* = 5). (c) Quantification of BL intensity at tumor sites in Panc02-Luc orthotopic pancreatic cancer-bearing mice (n = 5). (d) H&E staining images of orthotopic pancreatic tumors from mice following various treatments. (e) Photograph of tumors from orthotopic pancreatic cancer-bearing mice (*n* = 5). (f) Tumor weights of collected tumors (*n* = 5). (g) Survival curves of Panc02-Luc orthotopic pancreatic cancer-bearing mice following various treatments (*n* = 10). (h) BL imaging analysis of tumor metastasis in different organs for Panc02-Luc orthotopic pancreatic cancer-bearing mice. (i) Quantification of BL signal intensity in the intestines, stomach, kidney, lung, spleen, heart, and liver from mice in each group (*n* = 5). Data are expressed as mean ± SD. Statistical significance was determined using a two-tailed unpaired *t*-test, ∗p < 0.05, ∗∗p < 0.01 and ∗∗∗ (*p* < 0.001).

The therapeutic outcomes of deep-tissue orthotopic pancreatic cancer were evaluated following i.v. injection of SPN_NR_, SPN_N_H, and SPN_NR_H, combined with US irradiation (Fig. 6a). The antitumor and anti-metastasis efficacies were assessed using Panc02-Luc orthotopic pancreatic cancer mouse models the measurement of bioluminescence (BL) signals. D-Luciferin potassium salt, which is oxidized by luciferase in Panc02-Luc cells to generate BL signals, was used to monitor tumor growth and metastasis. After treatment with SPN_N_H + US, and SPN_NR_H + US, the BL signals were significantly reduced and nearly undetectable by day 14 (Fig. 6b). While the BL signals were also reduced in the SPN_NR_ + US group, which remained detectable after 14 days of treatment. In contrast, the BL signals increased in the other groups due to tumor growth. The lowest BL signal intensities were consistently observed in the SPN_NR_H + US group (Fig. 6c). On day 14, the BL signal intensity in the SPN_NR_H + US group was 7.0- and 6.1-fold lower than that in the SPN_NR_ + US and SPN_N_H + US groups, respectively.

Histological analysis revealed significant cell apoptosis in the SPN_NR_ + US, SPN_N_H + US, and SPN_NR_H + US groups (Fig. 6d), with the most pronounced apoptosis observed in the SPN_NR_H + US group. Photographs of excised tumors showed that the tumors in the SPN_NR_ + US, SPN_N_H + US, and SPN_NR_H + US groups were smaller than those in the PBS group (Fig. 6e). Notably, all tumors in the SPN_NR_H + US group were completely eradicated in five individual mice, while only one tumor and three tumors with smaller sizes were observed in the SPN_NR_ + US and SPN_N_H + US groups, respectively. The average tumor weight in the SPN_N_H + US group was as low as 0.03 g, significantly lower than that in the SPN_NR_ + US (0.14 g) and PBS (0.54 g) groups (Fig. 6f). The survival rate of mice in the SPN_NR_H + US group remained at 100 % after 25 days of treatment, compared to less than 70 % in the other groups (Fig. 6g). These results demonstrated the strong tissue penetration capability of US and highlight the superior antitumor therapeutic efficacy of SPN_NR_H + US.

Furthermore, the anti-metastasis efficacy was explored using BL imaging. Minimal BL signals were detected in the intestines, stomach, kidney, lung, spleen, heart, and liver in the SPN_NR_H + US group, whereas BL signals were observed in the liver, stomach, and intestines in the SPN_NR_, SPN_N_H, SPN_NR_H, PBS + US, SPN_NR_ + US, and SPN_N_H + US groups (Fig. 6h). The BL intensity in the heart, stomach, and intestines of the SPN_NR_H + US group was at least 3.2-, 5.6-, and 1.4-fold lower than that in the corresponding organs of the other groups, respectively (Fig. 6i). Furthermore, the BL signals and intensities in these tissues in the SPN_NR_H + US group were similar to those in healthy mice, indicating complete suppression of tumor metastasis.

### 3.6 In vivo ICD effect and immunological effect evaluation

**Fig. 7:**
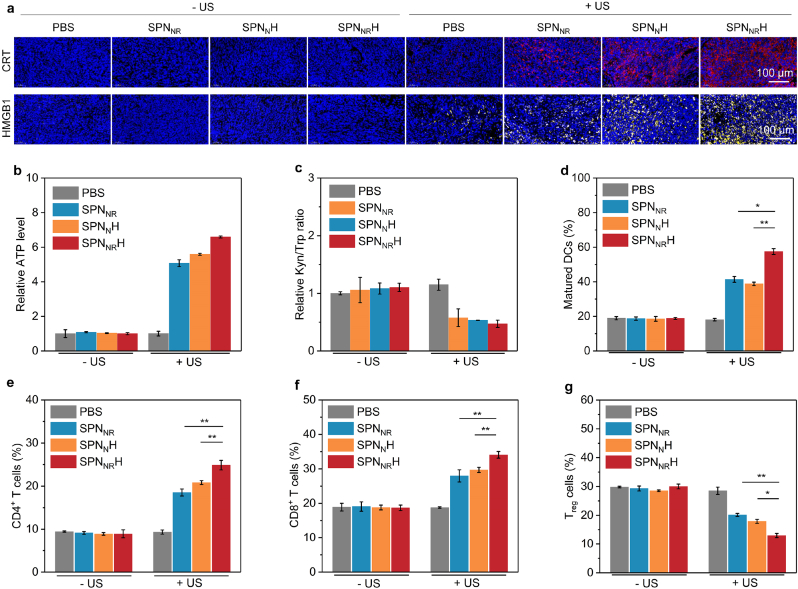
In vivo ICD effect and immunological effect evaluation. (a) Confocal fluorescence images of CRT and HMGB1 staining in orthotopic pancreatic tumors. (b) Quantification of ATP levels in each treatment group (*n* = 5). (c) Quantification of Kyn/Trp ratio in each treatment group (*n* = 5). (d) Quantification of matured DCs in each treatment group (*n* = 5). (e) Quantification of intratumoral CD4^+^T cells in each group (*n* = 5). (f) Quantification of intratumoral CD8^+^T cells in each treatment group (*n* = 5). (g) Quantification of intratumoral T_reg_ cells in each treatment group (*n* = 5). Data are expressed as mean ± SD. Statistical significance was determined using a two-tailed unpaired *t*-test, ∗ (*p* < 0.05) and ∗∗ (*p* < 0.01).

To further elucidate the immune-activating potential of SPN_NR_H, which has demonstrated remarkable antitumor efficacy, the alterations in the tumor immune microenvironment were meticulously assessed. The initial focus was on evaluating the ICD effect in orthotopic pancreatic tumors through immunofluorescence analysis. The findings revealed that pronounced fluorescence signals for CRT and HMGB1 were evident in the SPN_NR_ + US, SPN_N_H + US, and SPN_NR_H + US groups. In contrast, these signals were virtually undetectable in the remaining groups (Fig. 7a). Notably, the SPN_NR_H + US group exhibited the strongest CRT and HMGB1 staining signals. Specifically, the intensity of CRT fluorescence signals in the SPN_NR_H + US group was approximately 65.9 times higher than that in the non-US irradiation treatment group, 9.4 times higher than that in the PBS + US group, 2.2 times higher than that in the SPN_NR_ + US group, and 1.2 times higher than that in the SPN_N_H + US group (Fig. S4). Similarly, the highest HMGB1 fluorescence intensity was observed in the SPN_NR_H + US group (Fig. S5). Additionally, ATP levels in orthotopic pancreatic tumors were measured. While SPN_NR_, SPN_N_H, SPN_NR_H treatments alone did not significantly increase ATP levels, the combination of these nanostimulants with US irradiation led to a 5.1-fold increase in ATP levels for SPN_NR_ + US, a 5.6-fold increase for SPN_N_H + US, and a 6.6-fold increase for SPN_NR_H + US (Fig. 7b). These findings confirmed the potent ICD effect in the SPN_NR_H + US group.

To evaluate the modulation of the IDO immunosuppressive tumor microenvironment, the Kyn/Trp ratio in orthotopic pancreatic tumors was analyzed. The Kyn/Trp ratios in the US irradiation treatment groups were almost identical, whereas the ratios in the SPN_N_H + US and SPN_NR_H + US groups were significantly reduced (Fig. 7c). This indicates that following US irradiation, the SPN-based nanostimulants effectively degraded to release NLG919, thereby efficiently inhibiting IDO activity.

DCs play pivotal roles in cancer immunotherapy by facilitating antigen delivery and presentation, as well as activating T cells [32]. The content of mature DCs was only increased in the SPN_NR_ + US, SPN_N_H + US, and SPN_NR_H + US groups (Fig. S6). The mature DCs levels in these groups were 41.4 %, 38.8 %, and 57.5 %, respectively, compared to approximately 19.0 % in the remaining groups (Fig. 7d). To confirm the activation of antitumor immune responses, the intratumoral levels of T cells were then evaluated. while CD4^+^ T cell levels in SPNs groups did not significantly affect, all SPNs + US groups led to a noticeable increase (Fig. S6). Specifically, the CD4^+^ cell level in the SPN_NR_H + US group reached 23.6 %, higher than that in the SPN_NR_ + US (18.7 %) and SPN_N_H + US (20.3 %) groups (Fig. 7e). Similarly, the levels of CD8^+^ T cells within primary tumors were found to rise following SPNs + US treatments (Fig. S7). Notably, the CD8^+^ T cell number in SPN_NR_H + US group reached 33.2 %, which was higher than those in all other groups (Fig. 7f). T_reg_ cells play a crucial role in suppressing antitumor immune responses [54]. To further elucidate the immunological landscape within the tumor microenvironment, the intratumoral levels of T_reg_ cells were also analyzed. The treatments involving SPNs + US were found to down-regulate the levels of T_reg_ cells (Fig. S8). The lowest percentage of T_reg_ cells in tumor tissues was observed in the SPN_NR_H + US group (13.5 %), which was reduced by 2.3-fold (Fig. 7g). Consequently, the SPN_NR_H + US group exhibited significant suppression of T_reg_ cells, which in turn enhanced the functionality and activity of effector T cells.

### 3.7 Side effect evaluation

The safety profile of SPN_NR_H + US treatment was assessed in orthotopic pancreatic tumor-bearing mice. No significant changes in body weight were observed across various treatment groups (Fig. S9). Additionally, histological analysis via H&E staining revealed no evident damage to major organs between the SPN_NR_H + US group and the PBS control group (Fig. S10). Collectively, these results indicated that the SPN_NR_H + US combination treatment is both safe and effective for their in vivo anticancer applications.

## 4 Conclusion

In summary, we have developed ECM-degradable SPNs that facilitate precise drug delivery and extensive remodeling of the TME, thereby augmenting antitumor immunological effects in deep-seated orthotopic tumors. These SPNs incorporate HAase to degrade HA within the tumor ECM, diminishing the dense stroma and enhancing nanostimulants accumulation in tumors. Under US irradiation, the SPNs effectively generate ^1^O_2_ through the SDT effect of the PFODBT. The generated ^1^O_2_ not only induces ICD but also triggers the release of R848 and NLG919 from the ^1^O_2_-responsive nanostimulants, ensuring targeted delivery to tumor tissues. Additionally, ECM degradation improves immune cell infiltration into tumors. This multifaceted remodeling strategy using SPNs elicits a robust antitumor immunological response, leading to significant growth inhibition of deep-tissue orthotopic pancreatic tumors and resistance to tumor metastasis in mouse models. Our findings suggest that these ECM-degradable SPNs hold great promise for developing next-generation antitumor drugs, offering a novel approach to comprehensively remodel the TME for the immunotherapy of deep-seated orthotopic tumors.

## CRediT authorship contribution statement

**Meng Li:** Writing – original draft, Methodology, Investigation, Data curation. **Danling Cheng:** Writing – original draft, Methodology, Investigation, Data curation. **Yue Wang:** Methodology, Investigation. **Chongwen Xuan:** Methodology, Investigation. **Viktar Abashkin:** Writing – review & editing, Resources, Funding acquisition. **Jindong Xia:** Writing – review & editing, Resources, Funding acquisition. **Ling Ding:** Writing – review & editing, Supervision, Resources, Conceptualization. **Jingchao Li:** Writing – review & editing, Supervision, Resources, Funding acquisition, Conceptualization.

## Declaration of competing interest

The authors declare that they have no known competing financial interests or personal relationships that could have appeared to influence the work reported in this paper.

## Data Availability

Data will be made available on request.
