# Supplementary material for: Extracellular matrix-degradable polymer nanostimulants elicit potent immune responses in orthotopic pancreatic cancer via sono-activatable dual-drug synergism
Source: Mater Today Bio. 2025 Jun 6;33:101954. doi: 10.1016/j.mtbio.2025.101954 (PMC12178713; doi:10.1016/j.mtbio.2025.101954)
Supplement: Multimedia component 1 [file mmc1.docx]

**Supporting Information**

**Extracellular matrix-degradable polymer nanostimulants elicit potent immune responses in orthotopic pancreatic cancer via sono-activatable dual-drug synergism**

Meng Li^1#^, Danling Cheng^1#^, Yue Wang^2^, Chongwen Xuan^1^, Viktar Abashkin^3^*, Jindong Xia^2^*, Ling Ding^1^*, Jingchao Li^1^*

^1^ State Key Laboratory of Advanced Fiber Materials, College of Biological Science and Medical Engineering, Donghua University, Shanghai 201620, China

^2^ Department of Radiology, Songjiang Hospital Affiliated to Shanghai Jiao Tong University School of Medicine, Shanghai 201600, China

^3^ Institute of Biophysics and Cell Engineering of NASB, 27 Akademicheskaya St., 220072 Minsk, Belarus

^#^ These authors contributed equally to this work.

*Corresponding authors:

[jcli@dhu.edu.cn](mailto:jcli@dhu.edu.cn); [ling_ding@dhu.edu.cn](mailto:ling_ding@dhu.edu.cn); xiajd_21@163.com; [viktar.abashkin@gmail.com](mailto:viktar.abashkin@gmail.com)

**
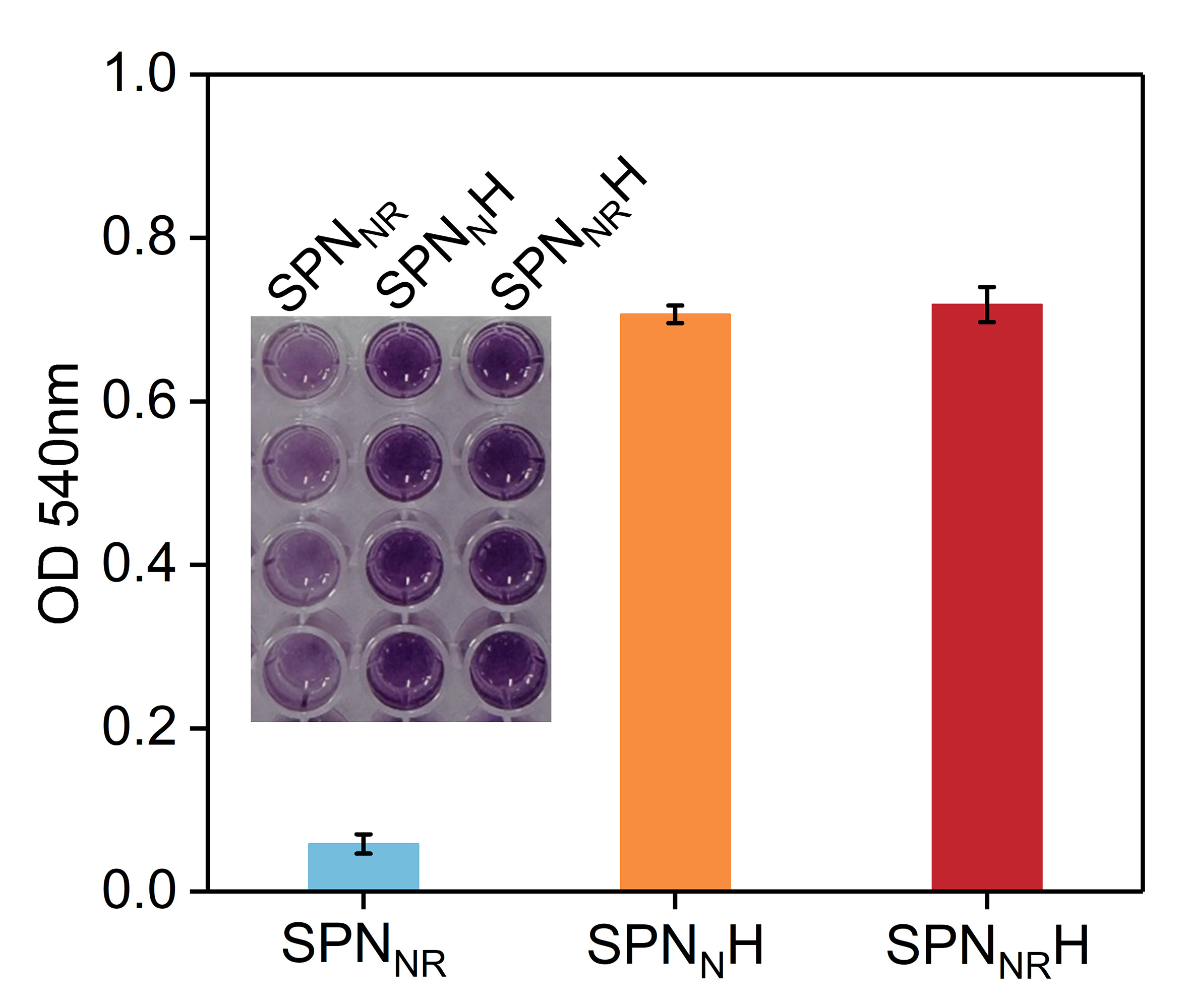
**

**Fig. S1.** BCA protein assay of SPN_NR_, SPN_N_H and SPN_NR_H (n = 3). Data are presented as means ± SD.

**
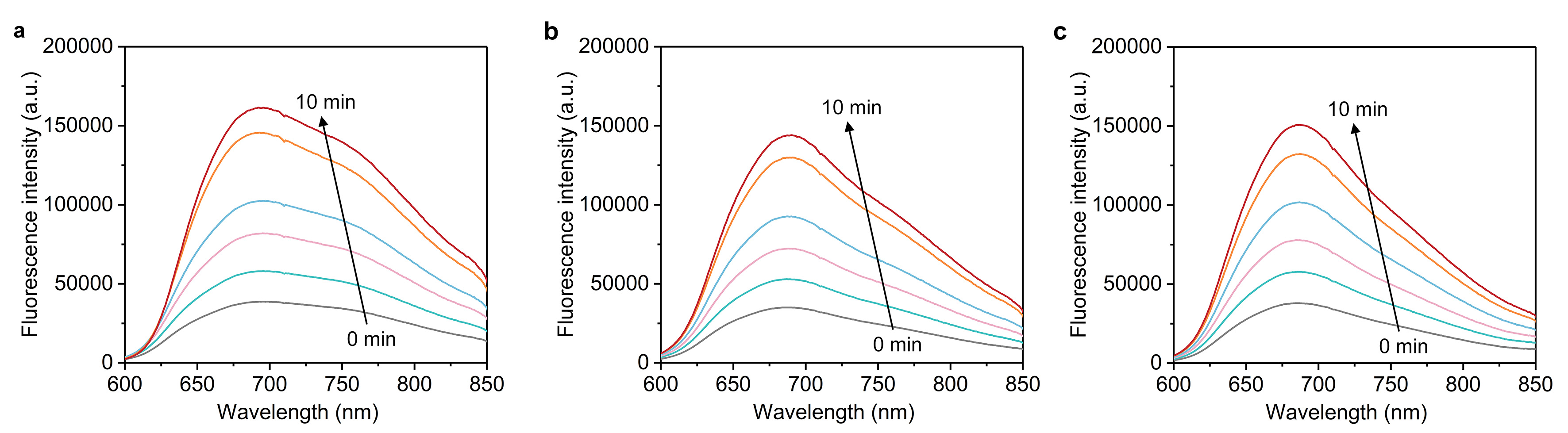
**

**Fig. S2.** Fluorescence spectra of SOSG in solutions containing (a) SPN_NR_, (b) SPN_N_H and (c) SPN_NR_H under US irradiation for different times.


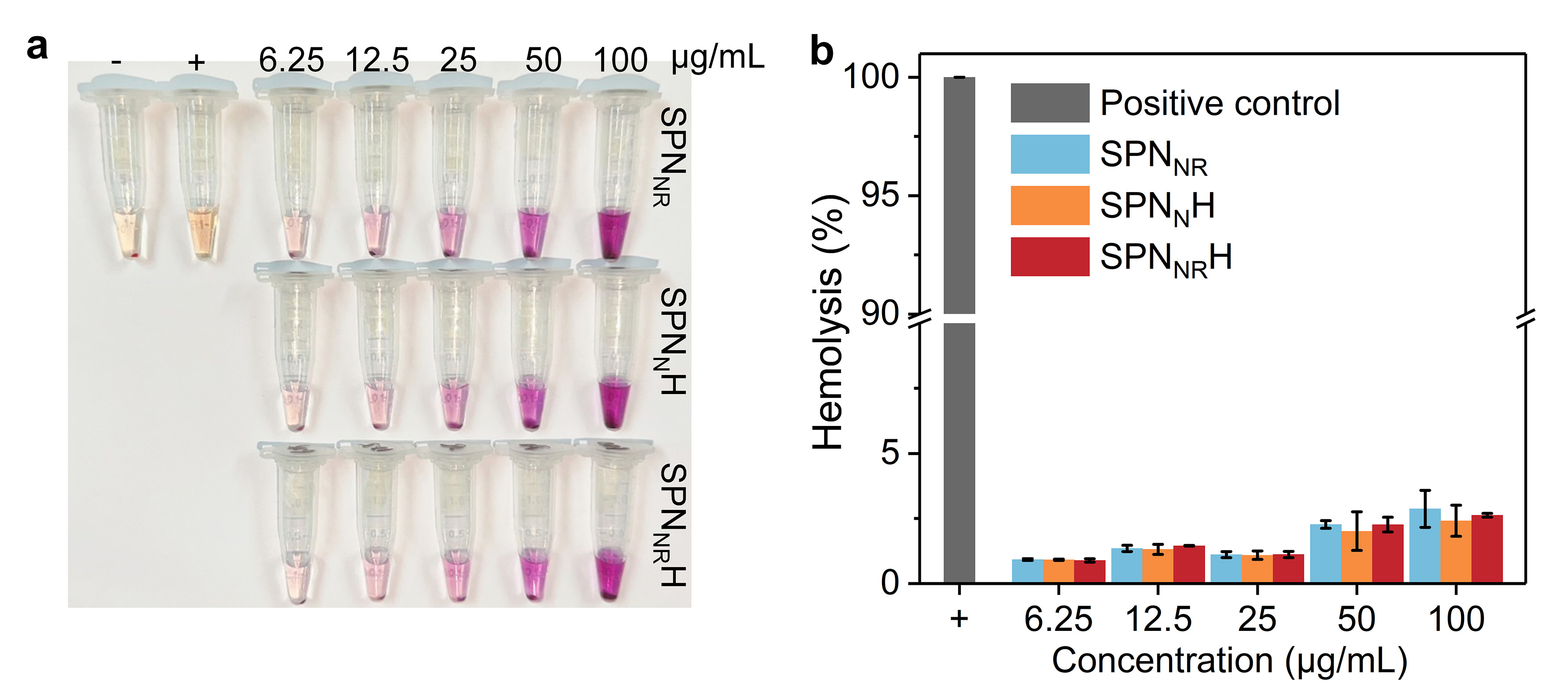


**Fig. S3.** (a) Hemolysis analysis of murine blood red cells after incubation with SPN_NR_, SPN_N_H and SPN_NR_H at different concentrations for 2 h. (b) Hemolysis percentages of murine blood red cells in different treatment groups (n = 5). Data are presented as means ± SD.

**
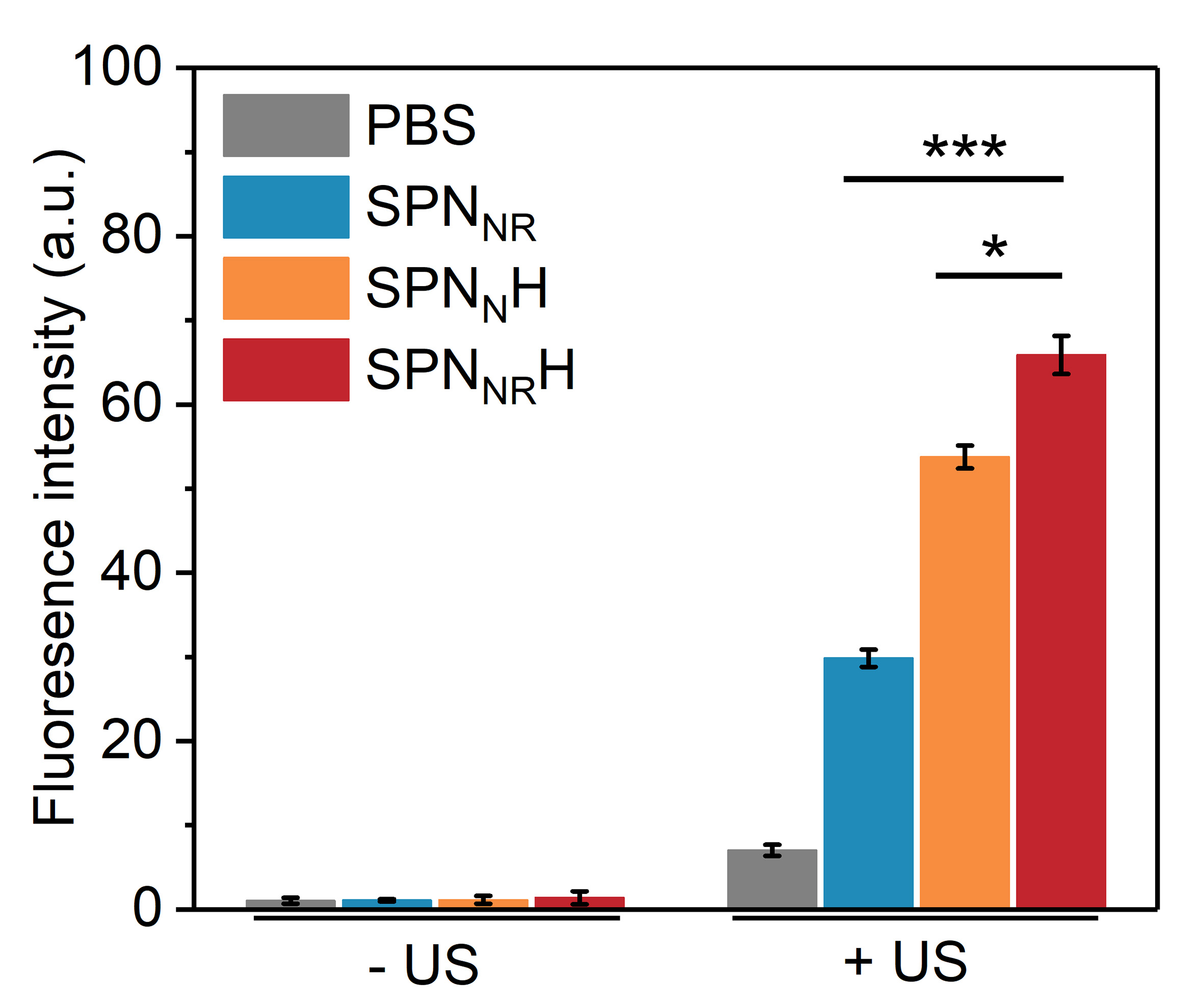
**

**Fig. S4.** CRT staining fluorescence intensity of tumors from various treated mice (n = 5). Data are presented as means ± SD. Statistical significance was determined using a two-tailed unpaired t-test, *p < 0.05 and *** (p < 0.001).

**
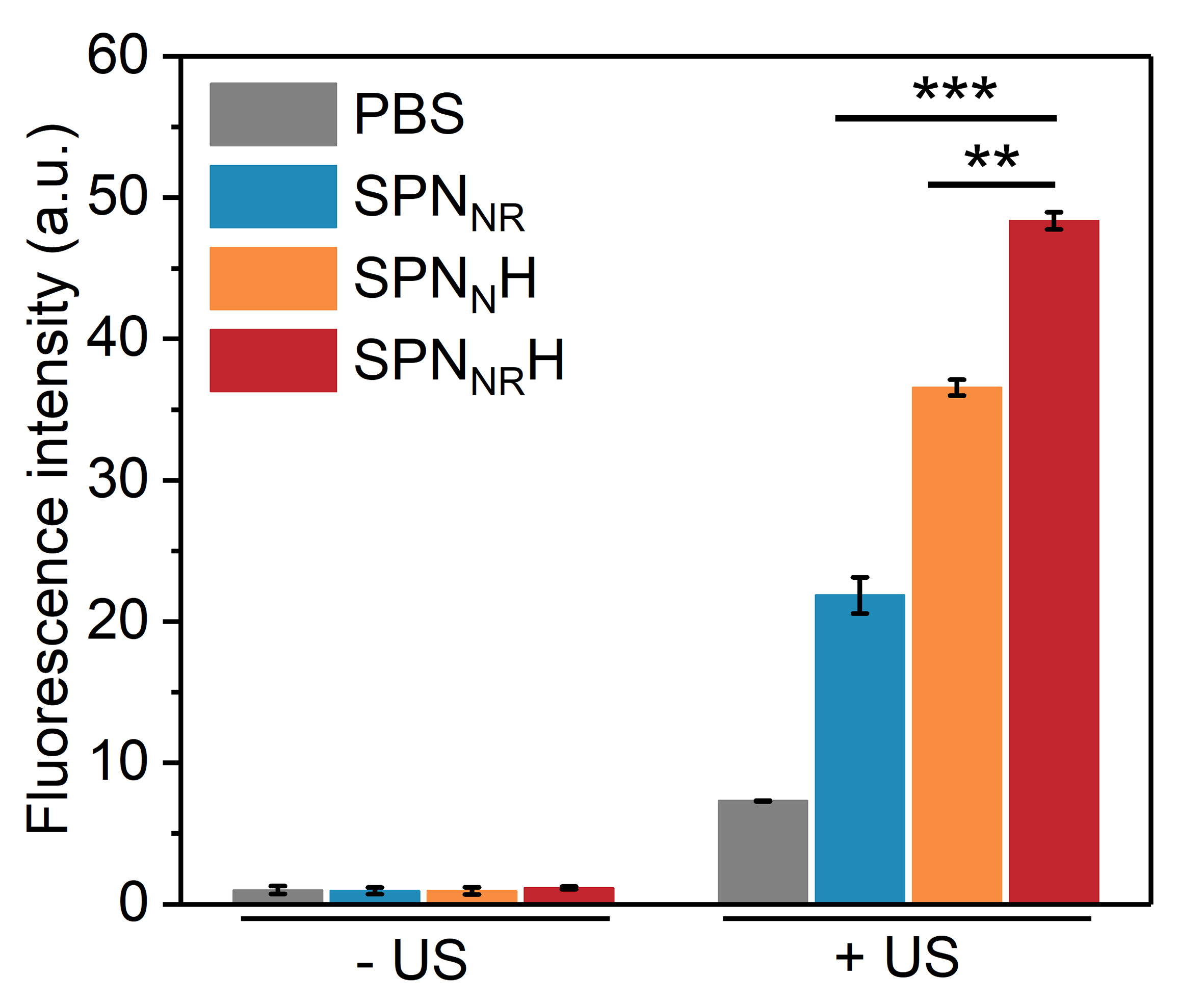
**

**Fig. S5.** HMGB1 staining fluorescence intensity of tumors from various treated mice (n =5). Data are presented as means ± SD. Statistical significance was determined using a two-tailed unpaired t-test, p < 0.01 and *** (*p* < 0.001).


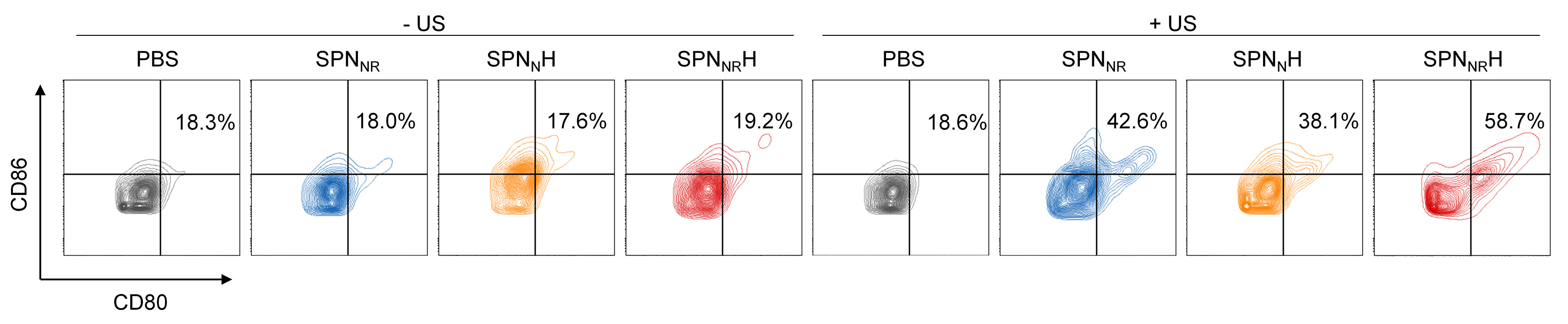


**Fig. S6.** Flow cytometry assay of matured DCs (CD80^+^CD86^+^) in tumor draining lymph nodes in various treatment groups.


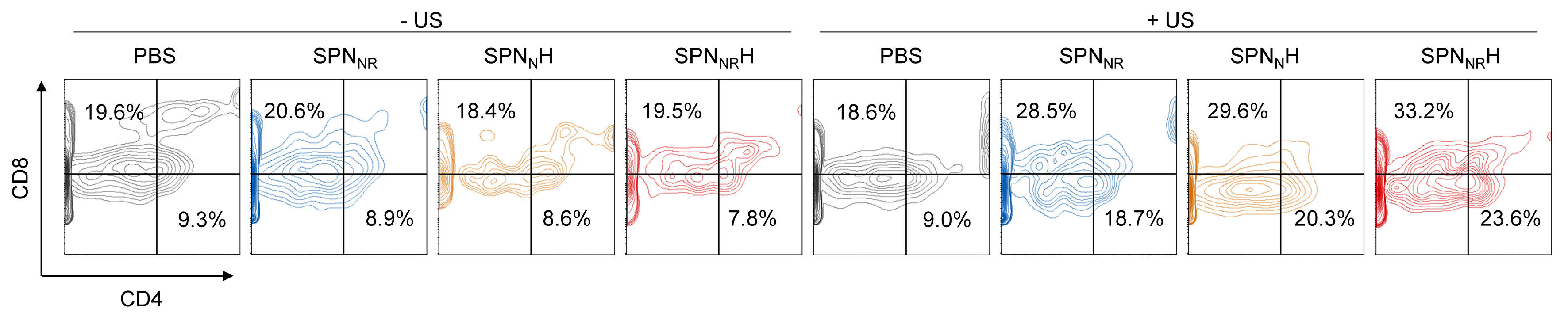


**Fig. S7.** Flow cytometry assay of CD4^+^ T cells and CD8^+^ T cells in tumors of mice with orthotopic pancreatic cancer in various treatment groups.


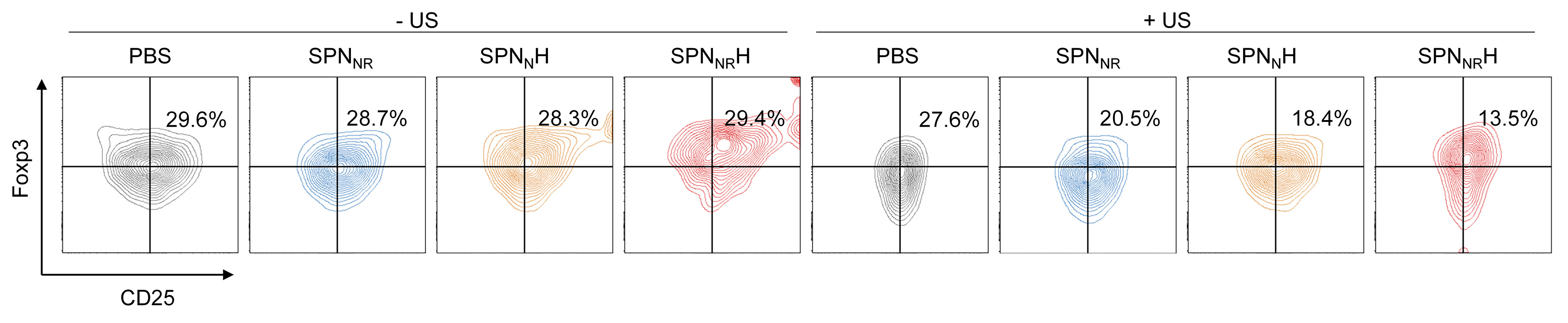


**Fig. S8.** Flow cytometry assay of Treg cells (CD25^+^Foxp3^+^) in tumors of mice with orthotopic pancreatic cancer in various treatment groups.

**
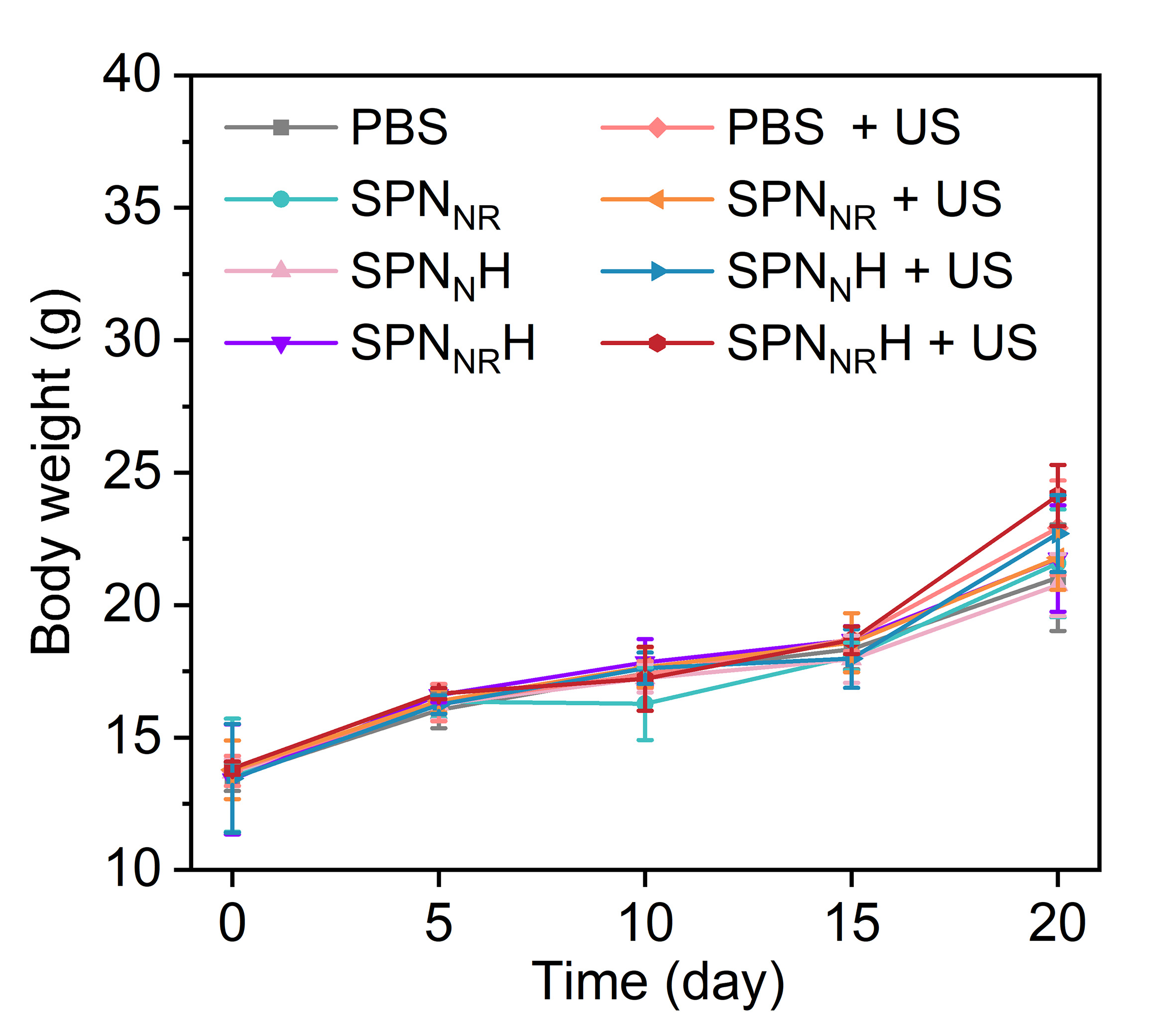
**

**Fig. S9.** The body weights of mice with orthotopic pancreatic cancer in different treatment groups (n = 5). Data are presented as means ± SD.


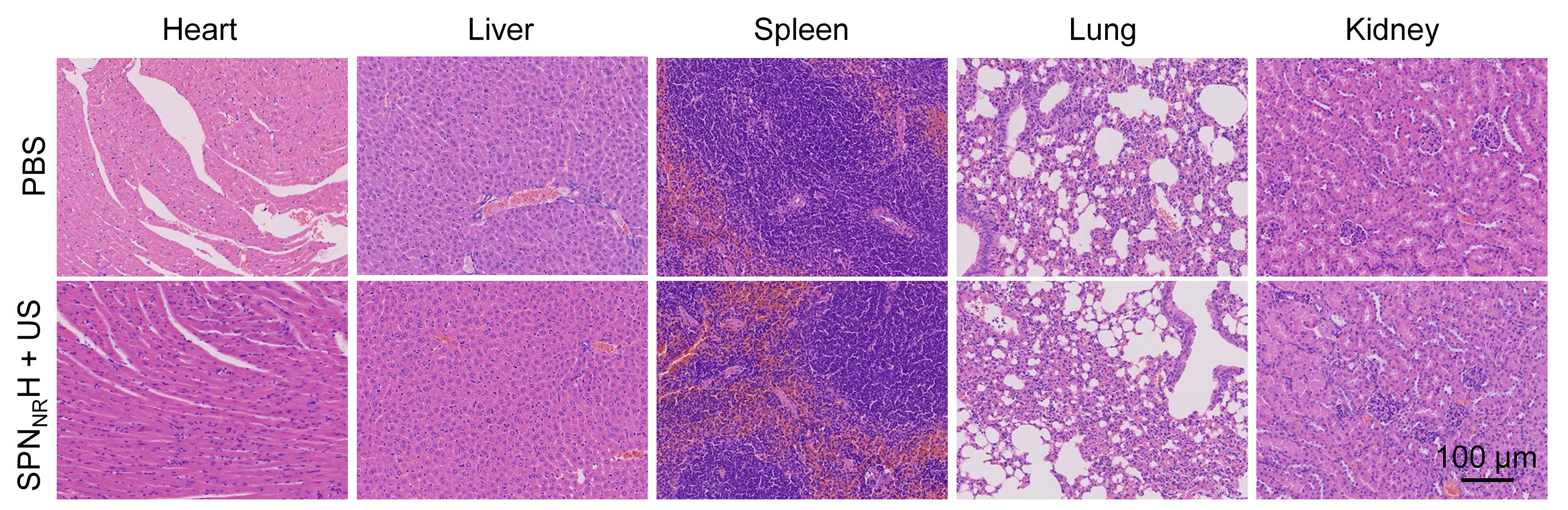


**Fig. S10.** H&E staining images of heart, liver, spleen, lung, kidney in PBS control and SPN_NR_H + US groups after treatments for 20 days.
